# Therapeutic gases and the Nrf2/Keap1 pathway: direct thiol modification *vs*. indirect modulation

**DOI:** 10.4103/mgr.MEDGASRES-D-26-00086

**Published:** 2026-08-26

**Authors:** Te Xiao, Chao Xia, Lisha Huang, Yanqi Li, Mengyu Liu, Pengxiang Zhao, Xuemei Ma, Fei Xie

**Affiliations:** 1 College of Chemistry and Life Science, Beijing University of Technology, Beijing, China; 2 Sichuan Academy of Traditional Chinese Medicine Sciences, Chengdu, Sichuan Province, China

**Keywords:** gasotransmitter, hydrogen sulfide, Keap1, mitochondrial quality control, molecular hydrogen, nitric oxide, Nrf2, persulfidation, redox signaling, S-nitrosylation

## Abstract

The nuclear factor erythroid 2-related factor 2/Kelch-like Ech-associated protein 1 (Nrf2/Keap1) pathway is a central homeostatic module that integrates oxidant, electrophilic, inflammatory, and metabolic signals into adaptive transcriptional programs. In parallel, nitric oxide (NO), hydrogen sulfide (H_2_S), and molecular hydrogen (H_2_) have emerged as therapeutically relevant gaseous mediators with distinct chemical identities, tissue distribution profiles, and pharmacological constraints. This review re-examines these gases through a single organizing question: how can highly dissimilar small molecules converge on the same redox-defense hub? We compare the direct cysteine chemistry of NO and H_2_S with the mainly indirect actions proposed for H_2_, and we use the Nrf2/Keap1 axis as the mechanistic backbone for that comparison. NO and H_2_S are best understood as redox-active signaling gases capable of covalent protein modification, whereas H_2_ appears to act largely by reshaping upstream oxidative events, mitochondrial electron leakage, membrane lipid oxidation products, and stress-responsive signaling states. Despite these differences, all three gases can feed into overlapping kinase pathways, mitochondrial quality-control networks, and cytoprotective gene programs. A second goal of this review is to move beyond single-gas narratives. We discuss the extent to which gas–gas crosstalk, especially between NO and H_2_S and the buffering role proposed for H_2_ under severe oxidative stress, may create systems-level convergence rather than simple additive antioxidant effects. We argue that the most productive conceptual model is not “one gas, one target”, but a context-dependent gas signaling network centered on Nrf2/Keap1, mitochondrial adaptation, and thiol chemistry. Finally, we assess the translational implications of this framework across ischemia–reperfusion injury, neurodegeneration, metabolic disease, and aging. We highlight the practical constraints that still limit clinical implementation, including dose windows, burst release, tissue targeting, biomarker selection, and the difficulty of tracking reversible cysteine modifications *in vivo*. By placing NO, H_2_S, and H_2_ into one comparative redox framework, this review aims to clarify what is established, what remains inferential, and where future combination strategies may realistically emerge.

## Introduction

Aerobic life depends on tightly regulated redox chemistry. Reactive oxygen species and reactive nitrogen species are not merely toxic by-products of metabolism; at physiological levels, they function as second messengers that shape proliferation, differentiation, immunity, metabolism, and tissue repair.^1^ Oxidative distress emerges when the rate, location, or persistence of reactive species exceeds the buffering capacity of antioxidant and repair systems, causing damage to lipids, proteins, and nucleic acids.^2^ Because this transition from signaling to injury is controlled rather than accidental, cellular defense systems must continuously discriminate between useful redox inputs and harmful ones.^3^

The best-studied coordinator of that discrimination is the nuclear factor erythroid 2-related factor 2 (Nrf2)/Kelch-like Ech-associated protein 1 (Keap1) pathway. Under homeostatic conditions, Keap1 acts as a cysteine-rich substrate adaptor that promotes cullin 3 (Cul3)-dependent ubiquitination and proteasomal turnover of Nrf2. Under oxidative or electrophilic challenge, strategically placed Keap1 cysteines change their reactivity and conformation, reducing Nrf2 degradation, enabling nuclear accumulation, and inducing a transcriptional program that includes antioxidant enzymes, glutathione synthesis machinery, detoxification systems, and genes involved in proteostasis, intermediary metabolism, and mitochondrial function.^3^,^4^ Because of this architecture, the pathway acts less like an on-off switch than like a biochemical sensor array that decodes a spectrum of redox states into adaptive transcriptional outputs.^5^,^6^

Therapeutic interest in gaseous small molecules grew from a different historical direction. Nitric oxide and hydrogen sulfide (H_2_S) entered mainstream biology as endogenous gasotransmitters, while molecular hydrogen was initially considered biologically inert until reports suggested selective protective effects against highly reactive oxidants.^7^,^8^,^9^ The three molecules differ profoundly in redox potential, bond chemistry, endogenous production, and pharmacokinetics. Nitric oxide (NO) is a radical, H_2_S is a nucleophilic/reducing sulfur species whose biological actions often proceed through reactive sulfur intermediates, and molecular hydrogen (H_2_) is a stable, nonpolar diatomic molecule with unusually high diffusibility but limited direct chemical reactivity.^10^,^11^ That contrast makes their repeated convergence on antioxidant and cytoprotective phenotypes scientifically intriguing rather than self-evident.

Most reviews still treat these gases separately. That approach is useful for cataloguing biosynthesis, canonical targets, and disease-specific studies, but it can obscure a deeper question: how can chemically distinct gases converge functionally on one stress-response hub? A comparative Nrf2-centered analysis provides a way to answer that question because Keap1-Nrf2 sits at the intersection of thiol chemistry, electrophile sensing, mitochondrial adaptation, inflammatory tone, and metabolic remodeling.^12^,^13^ It is therefore an ideal framework for distinguishing direct covalent mechanisms from indirect network effects.

This review develops four arguments. First, NO and H_2_S activate Nrf2 primarily through direct or near-direct mechanisms involving cysteine modification, although the exact residue preference and downstream consequences depend on the local redox context. Second, H_2_ appears to influence the pathway mainly through indirect remodeling of upstream oxidative events, mitochondrial electron leakage, and secondary electrophile formation, rather than through a defined direct receptor-like interaction.^11^,^14^ Third, all three gases converge on a shared layer of kinase signaling, proteostasis, and mitochondrial quality control that amplifies or stabilizes Nrf2 responses. Fourth, translational progress will likely depend less on maximizing gas dose and more on matching the right gas, timing, and delivery system to the right disease context. The purpose of this expanded review is therefore not simply to summarize gas biology, but to compare mechanistic logics and identify the degree to which multi-gas strategies are conceptually justified (**Figure 1**).

**Figure 1:**
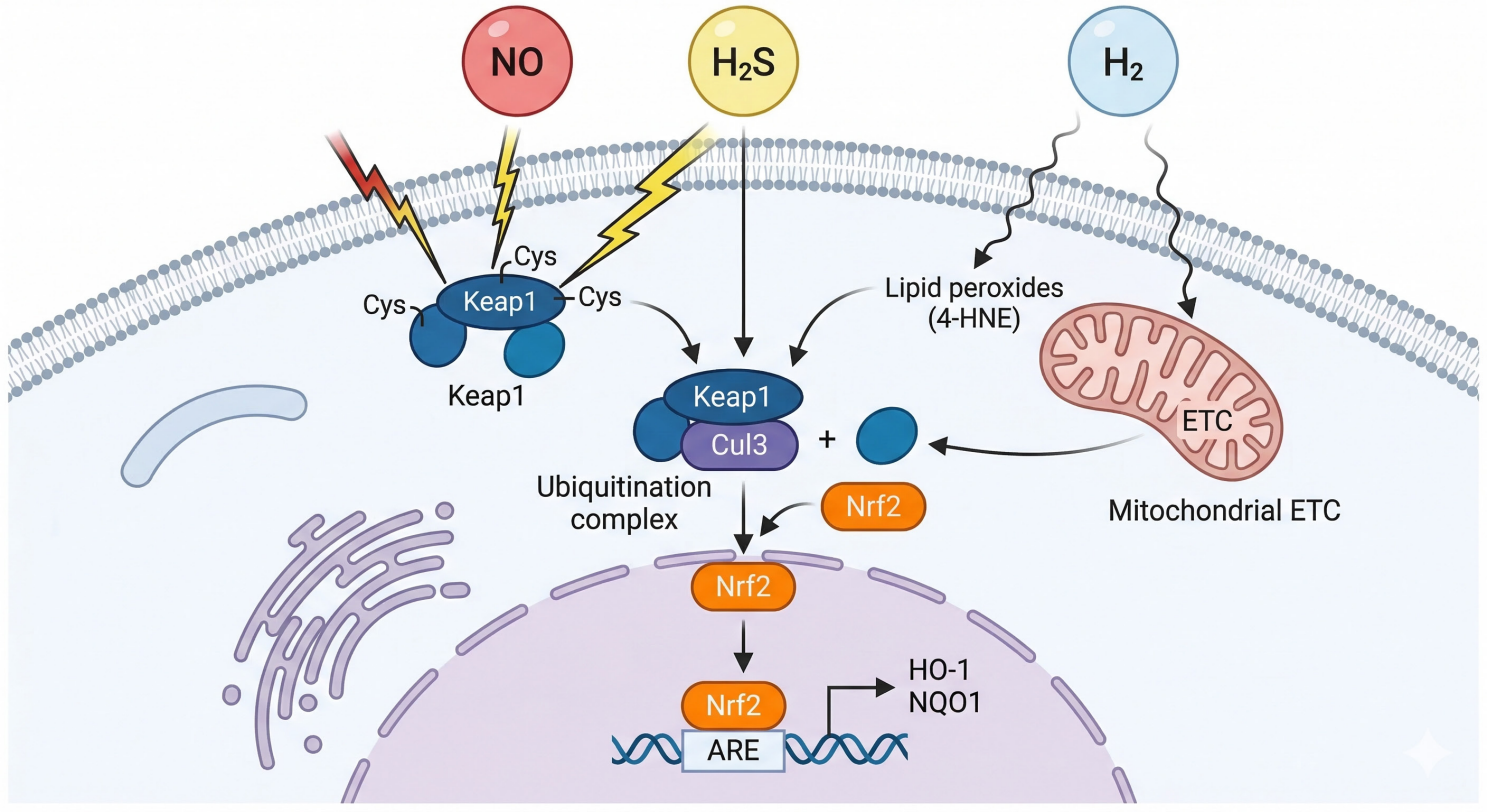
How do NO, H_2_S, and H_2_ converge on the Nrf2/Keap1 axis through direct or indirect routes? Created with BioRender.com. 4-HNE: 4-Hydroxynonenal; ARE: antioxidant response element; Cul3: cullin 3; Cys: cysteine; ETC: electron transport chain; H_2_: hydrogen; H_2_S: hydrogen sulfide; HO-1: heme oxygenase-1; Keap1: Kelch-like Ech-associated protein 1; NO: nitric oxide; NQO1: nad(p)h quinone oxidoreductase 1; Nrf2: nuclear factor erythroid 2-related factor 2.

## Search Strategy

A structured search of PubMed, Web of Science Core Collection, and Scopus was conducted from database inception to May 20, 2026. Search terms included “ Nrf2,” “NFE2L2,” “Keap1,” “nitric oxide,” “hydrogen sulfide,” “molecular hydrogen,” “gasotransmitter,” “S-nitrosylation,” “persulfidation,” “redox signaling,” “mitochondrial function,” “oxidative stress,” and “drug delivery.” Reference lists of relevant articles were also screened. English-language, peer-reviewed studies addressing NO, H2S, or H2 in relation to Nrf2/Keap1 signaling, redox regulation, mitochondrial function, disease applications, or delivery strategies were included. Duplicate records, conference abstracts, editorials, non-English publications, and studies lacking mechanistic or translational relevance were excluded. Recent studies were prioritized, while landmark earlier publications were retained when necessary.

## The Nrf2/Keap1 Axis Is a Cysteine-Sensitive Signaling Hub

Nrf2 belongs to the Cap‘n’collar basic leucine zipper family and orchestrates a transcriptional network centered on antioxidant response elements. The importance of the pathway is no longer confined to classic phase II detoxification. Nrf2 also influences glutamine utilization, nicotinamide adenine dinucleotide phosphate production, iron handling, proteasomal function, autophagy, mitochondrial biogenesis, and inflammatory set points.^3^,^4^

The canonical mechanism of pathway repression has become a paradigmatic example of thiol-based stress sensing. Keap1 is a dimeric protein containing an N-terminal BTB domain, an intervening region rich in reactive cysteines, and a Kelch domain that binds Nrf2. In basal conditions, one Keap1 dimer engages a single Nrf2 molecule through high-affinity and low-affinity motifs, presenting Nrf2 for Cul3-mediated ubiquitination. Electrophiles or oxidants alter selected Keap1 cysteines and disrupt this efficient turnover cycle, allowing newly synthesized Nrf2 to escape degradation and accumulate in the nucleus.^3^,^4^,^5^

The so-called cysteine code is especially relevant for gas biology. Keap1 contains many cysteines, but they are not equivalent. Cys151 is particularly associated with sensing reactive electrophiles and changes near the BTB-Cul3 interface, whereas Cys273 and Cys288 often emerge in models involving larger conformational effects on substrate orientation and repression efficiency.^3^,^4^,^5^

A comparative review centered on NO, H_2_S, and H_2_ must also consider noncanonical activation. p62/sequestosome1 can compete with Nrf2 for Keap1 binding when autophagic flux changes, thereby stabilizing Nrf2 independently of direct cysteine oxidation. In addition, glycogen synthase kinase 3 (GSK-3) can promote β-transducin repeatcontaining protein-dependent Nrf2 degradation through a Keap1-independent route, while adenosine monophosphateactivated protein kinase (AMPK) and other kinases modulate Nrf2 localization and activity under nutrient and energetic stress.^15^,^16^,^17^

The translational consequence of this architecture is important. Nrf2 activation is beneficial in many acute injury settings, but indiscriminate or chronic activation can also create liabilities, including reductive imbalance, altered immune tone, and in some contexts support of malignant cell survival.^4^,^6^ Therefore, the therapeutic question is not whether gas-mediated Nrf2 activation occurs, but under what timing, dose, and disease stage it helps rather than harms.

## Nitric Oxide and Hydrogen Sulfide Direct Modulate Nrf2/Keap1 Axis

NO is generated endogenously by nitric oxide synthases, diffuses rapidly across membranes, and participates in vasodilation, neurotransmission, platelet regulation, and host defense. Yet the same molecule can contribute to nitrative injury when produced in excess or when rapidly converted to secondary species such as peroxynitrite.^7^,^18^ In Nrf2, the most direct mechanism is S-nitrosylation of reactive cysteines on Keap1. NO and NO-derived nitrosating equivalents can modify Keap1, weaken its repression of Nrf2, and promote transcription of cytoprotective genes.^19^,^20^ NO rarely acts alone in biological systems. Crosstalk with phosphatidylinositol 3kinase (PI3K)/protein kinase B (Akt), extracellular signalregulated kinase 1/2, and AMPK can amplify Nrf2 stabilization, while changes in cyclic guanosine monophosphate signaling, endothelial shear stress responses, and mitochondrial respiration alter Nrf2.^13^,^21^ Consequently, the protective phenotype attributed to NO often represents the sum of direct S-nitrosylation, kinase-mediated signal propagation, and vascular-hemodynamic improvement rather than one isolated molecular event. NO promotes S-nitrosylation-dependent relaxation of Keap1 repression than implying that dissolved NO directly binds a unique molecular receptor on Keap1.^19^,^20^ The biological meaning of NO-dependent Nrf2 activation also depends on concentration and timing. At moderate levels, the pathway contributes to vascular protection, anti-inflammatory feedback, and adaptation to transient stress. Under severe inflammatory or ischemia-reperfusion conditions, however, simultaneous superoxide production shifts NO chemistry toward peroxynitrite formation, protein tyrosine nitration, and mitochondrial dysfunction.^18^ This complexity explains why NO remains a double-edged therapeutic candidate. Very short half-life, steep dose-response behavior, high susceptibility to oxidative consumption, and systemic hypotensive risk all constrain clinical implementation.

H_2_S is often described as a gasotransmitter analogous to NO, yet its chemistry is distinct. H_2_S exists in equilibrium with hydrosulfide ion (HS−) and participates in a broader reactive sulfur species network rather than acting solely as a free dissolved gas. Its effects depend on enzymatic production, mitochondrial sulfide oxidation, local pH, metal centers, disulfides, and sulfur-transfer reactions.^8^,^9^,^10^ These features make H_2_S particularly suited to modifying thiols and sulfur-rich protein microenvironments.

Several studies support the view that H_2_S donors or endogenous H_2_S-generating systems promote Keap1 persulfidation, reduce repression of Nrf2, and enhance antioxidant gene expression.^22^ In acute injury settings, controlled H_2_S delivery can lower energy demand, attenuate oxidative bursts, preserve endothelial function, and indirectly sustain Nrf2-dependent defense programs. In chronic settings, H_2_S has been linked to vascular homeostasis, anti-inflammatory feedback, and improved redox resilience.^8^,^21^ For translational design, H_2_S thus appears less as a generic antioxidant and more as a controllable thiol-modifying cue with a relatively strong mechanistic bridge to Nrf2/Keap1.

NO and H_2_S react chemically and influence each other’s biosynthesis, degradation, and signaling outputs. The resulting network includes hybrid reactive sulfur-nitrogen species, such as HSNO and SSNO−, along with downstream shifts in nitrosothiol and persulfide pools.^1^,^23^

HSNO has attracted particular attention because it is small, membrane permeable, and capable of transferring nitroso or sulfur-related reactivity depending on context.^23^,^24^ Although some intermediate species are transient and difficult to quantify *in vivo*, the broader conclusion is well supported: NO-H_2_S interactions generate chemically distinct products that can redistribute signaling competence across membranes and protein thiols. In practical terms, this means that co-exposure to NO and H_2_S is not simply additive; it can qualitatively change the redox species landscape encountered by Keap1 and other signaling proteins (**Figure 2**).

**Figure 2:**
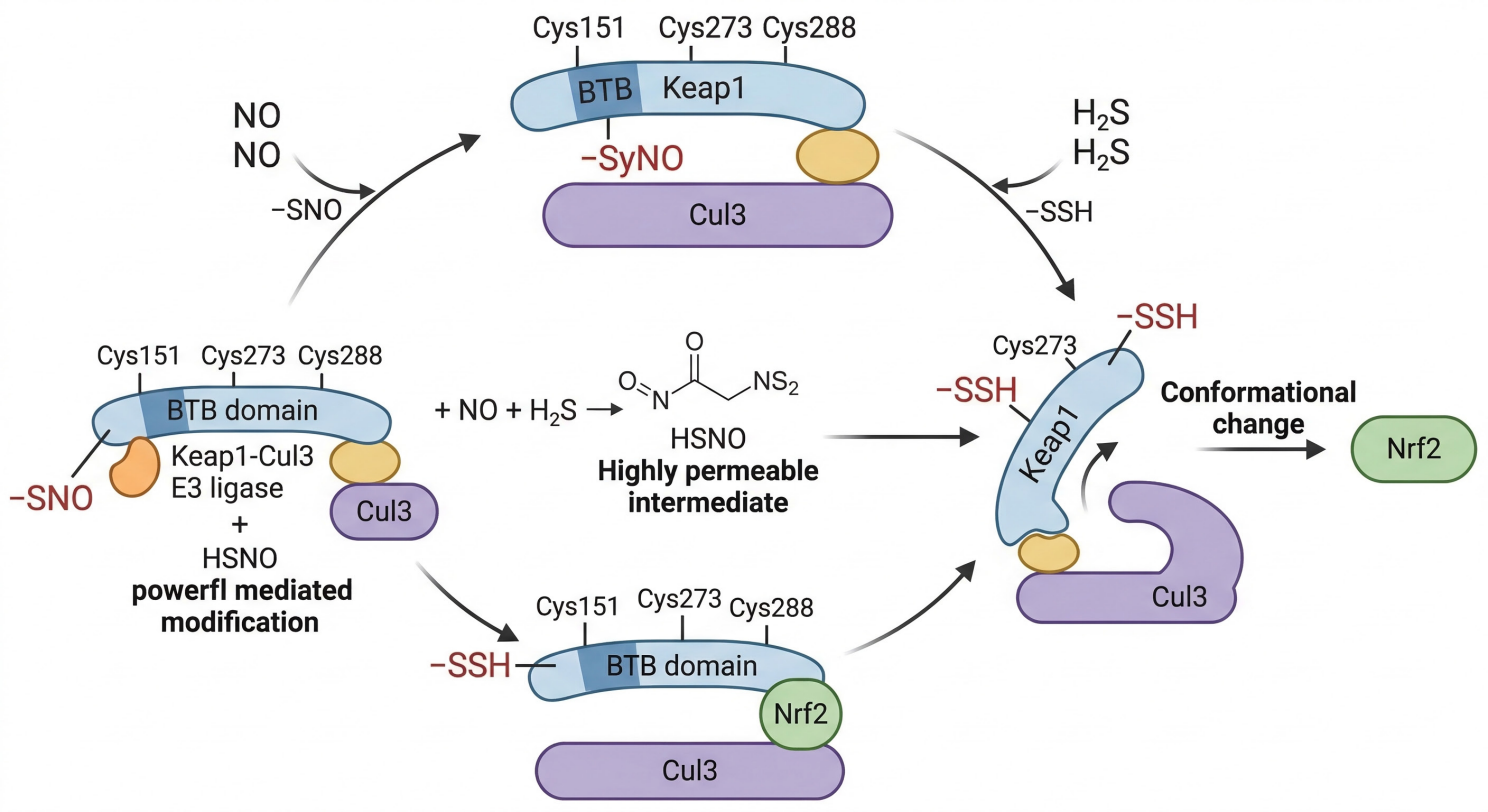
Direct covalent regulation of Keap1 by NO and H_2_S. Created with BioRender.com. BTB: Bric-a-brac, tramtrack, and broad complex; Cul3: cullin 3; H_2_S: hydrogen sulfide; HSNO: nitrosothiol; Keap1: Kelch-like Ech-associated protein 1; NO: nitric oxide; Nrf2: nuclear factor erythroid 2-related factor 2; SSH: S-sulfhydration.

For Nrf2, the important point is not that every proposed intermediate has been definitively mapped *in vivo*, but that NO and H_2_S can reshape each other’s access to the thiol network. Nitrosylation may be converted, buffered, or redirected by sulfur chemistry, while persulfidation states can alter sensitivity to additional oxidation or nitrosation.^10^,^25^

The vascular system provides a particularly intuitive example. Endothelial health depends on balanced NO bioavailability, mitochondrial competence, and suppression of chronic oxidative stress. H_2_S has been reported to support endothelial function and NO signaling partly by lowering oxidative burden and influencing endothelial NO synthase coupling, whereas NO can modulate enzymes involved in sulfur metabolism and vascular tone.^21^,^25^ Nrf2 becomes a systems integrator in this setting because it links both gas pathways to antioxidant transcription, glutathione homeostasis, and endothelial stress adaptation. At the same time, most evidence remains preclinical, mechanistically heterogeneous.

## Molecular Hydrogen Indirectly Regulates Nrf2/Keap1 Axis

H_2_ is electrically neutral, nonpolar, and kinetically stable under physiological conditions. It lacks the overt thiol-reactive chemistry that makes NO and H_2_S natural candidates for Keap1 modification. Yet a substantial literature describes anti-inflammatory, anti-apoptotic, and antioxidant-like effects of H_2_ across organ injury, neurological disease, metabolic dysfunction, and exercise-related stress.^9^,^14^,^26^

One influential hypothesis is that H_2_ attenuates runaway free-radical chain reactions in membranes while allowing limited generation of secondary electrophilic mediators, including oxidized phospholipid derivatives and 4-hydroxynonenal, that can themselves activate adaptive signaling programs.^27^,^28^ In this model, H_2_ does not directly oxidize or reduce Keap1. Instead, it changes the quality and quantity of upstream oxidative events, thereby shifting the electrophile landscape presented to the Keap1 sensor system.

Since H_2_ diffuses rapidly into subcellular compartments, it is well positioned to influence local redox dynamics at the level of the electron transport chain. Several authors have proposed that H_2_ reduces pathological electron leakage, improves respiratory efficiency under stress, and secondarily alters NAD^+^/NADH balance, Sirt1 activity, and downstream transcriptional programs linked to stress resistance and mitochondrial biogenesis.^11^,^14^,^29^

The link to Nrf2 is therefore probably indirect but biologically coherent. If H_2_ lowers pathological electron leakage, reshapes lipid oxidation products, or improves energetic signaling, it can influence AMPK, PI3K/Akt, GSK-3, and autophagy-related pathways that determine Nrf2 stabilization and transcriptional output.^15^,^16^,^17^

An additional reason H_2_ remains therapeutically attractive is its unusually broad safety window. Unlike NO and H_2_S, H_2_ does not usually exhibit a narrow biphasic toxicity threshold under normobaric therapeutic conditions, and it can be administered through inhalation, hydrogen-rich water, hydrogen-rich saline, or emerging carrier platforms (**Figure 3** and **Table 1**).^26^,^30^,^31^

**Figure 3:**
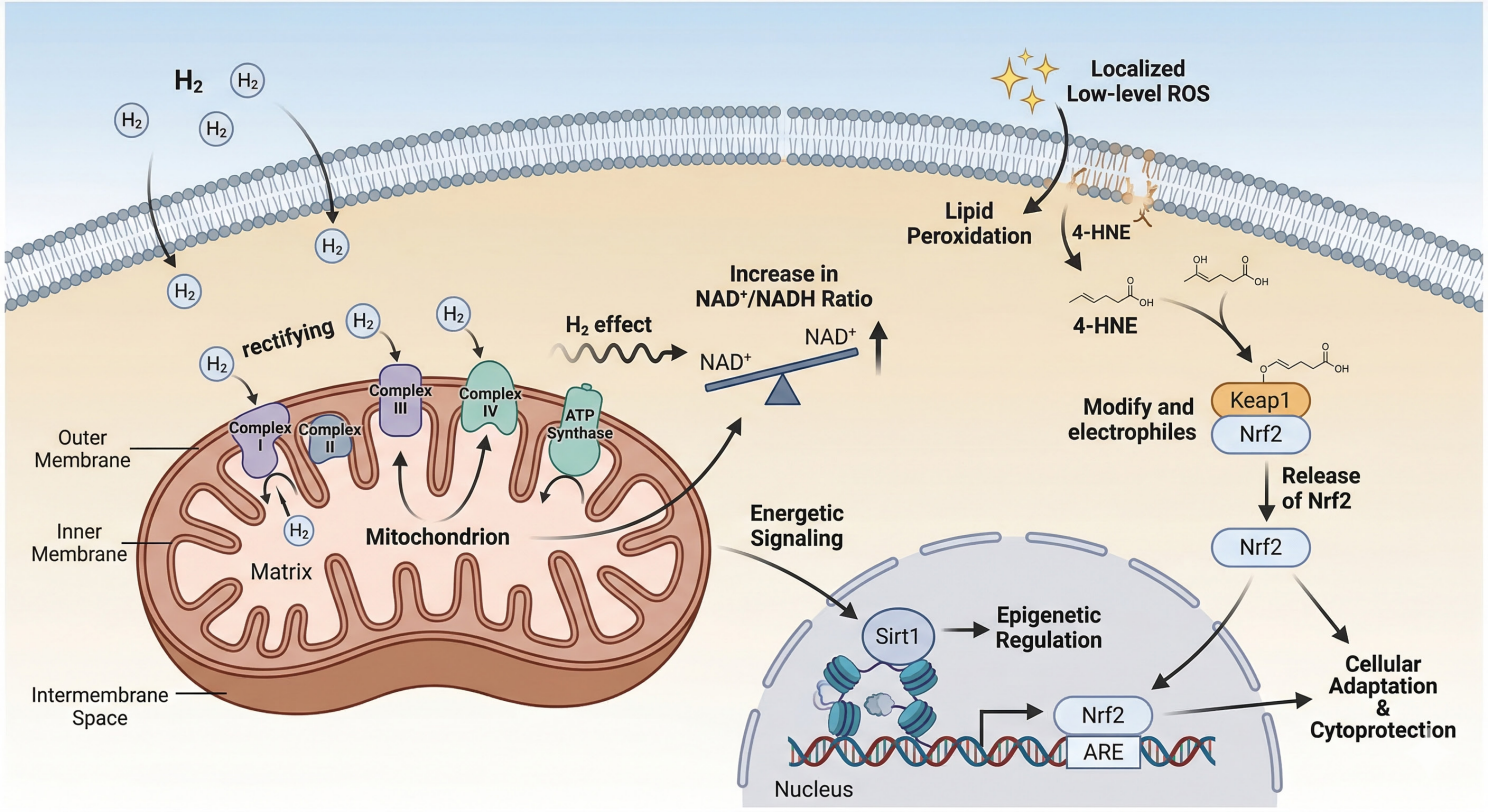
Molecular hydrogen indirectly regulates Nrf2/Keap1 axis. Created with BioRender.com. 4-HNE: 4-Hydroxynonenal; ARE: antioxidant response element; ATP: adenosine triphosphate; H_2_: hydrogen; Keap1: Kelch-like Ech-associated protein 1; NAD^+^: nicotinamide adenine dinucleotide (oxidized form); NADH: nicotinamide adenine dinucleotide (reduced form); Nrf2: nuclear factor erythroid 2-related factor 2; Sirt1: sirtuin 1.

**Table 1:** Comparative mechanistic features of NO, H_2_S, and H_2_ in relation to Nrf2/Keap1 signaling

Gas molecule	Dominant chemical identity	Nrf2 activation route	Representative Keap1-sensitive chemistry	Shared upstream signaling	Key translational limitation
NO	Radical gasotransmitter with rapid diffusion and high context dependence	Direct or near-direct S-nitrosylation-dependent relief of Keap1 repression plus kinase amplification	Nitrosylation focused Cys151-sensitive signaling environments	PI3K/Akt, ERK1/2, AMPK, endothelial signaling	Very short half-life; oxidative consumption; narrow dose window; hypotension risk
H_2_S	Reactive sulfur species network centered on sulfide/persulfide chemistry	Direct persulfidation of Keap1 and related thiol relays; mitochondrial and vascular support	Persulfidation involving Cys151/Cys273/Cys288-sensitive domains in different models	PI3K/Akt, ERK1/2, AMPK, sulfur-metabolic signaling	Burst release and mitochondrial toxicity at high dose; donor chemistry matters
H_2_	Chemically mild, nonpolar, highly diffusible molecule	Mainly indirect activation through remodeling of oxidative propagation, mitochondrial function, and secondary electrophiles	No established direct Keap1 adduct; likely mediated through lipid oxidation products, kinase shifts, and autophagy/metabolic signaling	PI3K/Akt, AMPK, GSK-3/β-TrCP, autophagy-related pathways	Mechanism remains inferential; tissue concentration and retention vary by delivery route

Akt: Protein kinase B; AMPK: adenosine monophosphate-activated protein kinase; ERK1/2: extracellular signal-regulated kinase 1/2; GSK-3: glycogen synthase kinase 3; H_2_: hydrogen; H_2_S: hydrogen sulfide; Keap1: Kelch-like Ech-associated protein 1; NO: nitric oxide; Nrf2: nuclear factor erythroid 2-related factor 2; PI3K: phosphatidylinositol 3-kinase; β-TrCP: β-transducin repeat-containing protein.

## Nrf2/Kaep1 Axis as a Shared Signal Axis for Gas Treatment

While direct Keap1 modification is strong for NO and H_2_S, H_2_ engages this redox layer indirectly by reshaping the membrane lipid oxidation landscape. This shifts the production of secondary mediators, such as 4-hydroxynonenal and oxidized phospholipids, which then act as the proximal triggers for Keap1. Importantly, NO and H_2_S also intersect with these networks, generating reactive species like nitrated fatty acids, making lipid-derived electrophiles a shared mechanistic bridge to Nrf2 across all three gases. PI3K/Akt, extracellular signalregulated kinase 1/2, p38 mitogen-activated protein kinase, and AMPK repeatedly appear across gas studies as amplifiers or stabilizers of Nrf2 responses.^13^,^17^

The PI3K/Akt-GSK-3 axis is especially important because it links survival signaling, nutrient state, and oxidative stress. Inhibition of GSK-3 reduces β-transducin repeatcontaining protein-mediated Nrf2 degradation, thereby complementing canonical Keap1 disengagement.^16^ NO can engage survival kinases through endothelial and receptor-linked pathways; H_2_S influences kinase signaling through both redox and mitochondrial routes; and H_2_ is often proposed to stabilize Nrf2 chiefly by improving energetic signaling and limiting maladaptive reactive oxygen species (ROS) amplification.^8^,^11^,^21^

p62 accumulation or altered autophagic flux can sequester Keap1 and produce noncanonical Nrf2 activation.^15^ At the same time, Nrf2 feeds back on proteasomal capacity, glutathione synthesis, and detoxification pathways that help maintain protein quality control.

Nrf2 regulates genes linked to nicotinamide adenine dinucleotide phosphate generation, iron-sulfur balance, mitochondrial biogenesis, and in some contexts mitophagy.^32^,^33^ Gas-mediated Nrf2 activation therefore has consequences beyond antioxidant enzyme induction. NO modulates respiration and vascular oxygen delivery, H_2_S interacts directly with mitochondrial sulfur metabolism and cytochrome c oxidase, and H_2_ is repeatedly associated with improved mitochondrial efficiency or reduced injury propagation.^8^,^11^

NO and H_2_S exert specific, well-characterized effects on transcription factors and chromatin-associated proteins through direct nitrosylation and persulfidation, respectively.^10^,^20^,^27^

Taken together, the question is whether distinct upstream chemistries can stabilize overlapping adaptive attractor states (stable, self-sustaining configurations of cellular networks) centered on Nrf2, mitochondrial resilience, and proteostasis. Current evidence supports broader systems-level convergence (**Figure 4**).

**Figure 4:**
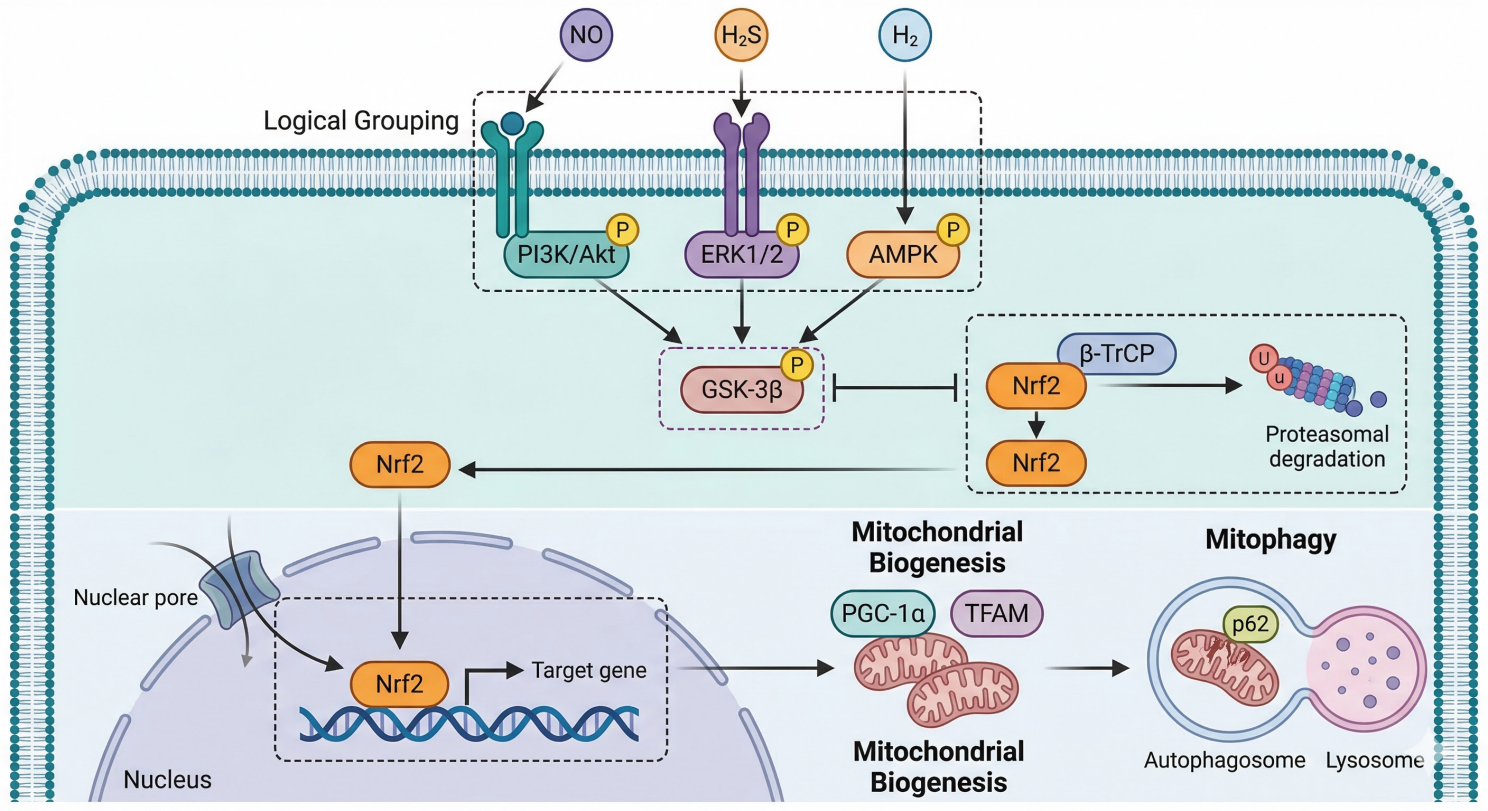
Nrf2/Kaep1 axis as a shared signal axis for gas treatment. Created with BioRender.com. Akt: Protein kinase B; AMPK: adenosine monophosphate-activated protein kinase; ERK1/2: extracellular signal-regulated kinase 1/2; GSK-3β: glycogen synthase kinase 3β; H_2_: hydrogen; H_2_S: hydrogen sulfide; Keap1: Kelch-like Ech-associated protein 1; NO: nitric oxide; Nrf2: nuclear factor erythroid 2-related factor 2; p62: sequestosome-1; PGC-1α: peroxisome proliferator-activated receptor gamma coactivator 1α; PI3K: phosphatidylinositol 3-kinase; TFAM: mitochondrial transcription factor A; β-TrCP: β-transducin repeat-containing protein.

## Nrf2-Centered Gas Framework

Nrf2/Keap1 is a central node in this network because it translates all three input classes into a common adaptive language: transcription of antioxidant, detoxification, metabolic, and proteostatic genes. However, the network model also emphasizes asymmetry. The three gases are not interchangeable. NO is fast, locally intense, and highly context sensitive. H_2_S is chemically rich but dose constrained. H_2_ is pharmacologically forgiving but mechanistically diffuse. As a result, the practical value of a combination strategy is likely to depend on whether the clinical problem requires rapid local signaling, sustained thiol protection, broad tissue penetration, or some temporally staged combination of these features. These are plausible, biologically grounded strategies, but they remain hypotheses or early translational concepts in most disease settings.

## Nrf2-Centered Gas Framework and Diseases

Ischemia-reperfusion injury remains one of the clearest contexts in which an Nrf2-centered gas framework has translational appeal. Ischemia depletes ATP and primes mitochondria for burst ROS generation upon reperfusion, while microvascular dysfunction and inflammation further amplify injury.^34^ H_2_S has been explored as a metabolic suppressor or preconditioning signal during the ischemic phase, NO as a modulator of perfusion and endothelial function during reperfusion, and H_2_ as a broadly distributed attenuator of oxidative propagation with a favorable safety profile.^8^,^30^ Within an Nrf2 framework, these interventions may act at different temporal stages of the same injury cascade rather than competing for the same mechanistic niche.

In neurodegenerative diseases, the appeal of H_2_ is strengthened by its diffusibility and ability to reach the central nervous system via the routes of simple administration.^21^,^35^ Yet, NO and H_2_S pathways are also deeply involved in neurovascular coupling, inflammation, and synaptic redox regulation. The challenge is that the brain is exquisitely sensitive to both oxidative stress and inappropriate nitrosative or sulfide exposure. A network view suggests that broad, low-risk H_2_ exposure may be best positioned as a background modulator, whereas NO or H_2_S interventions may require targeted or donor-based delivery to avoid systemic liabilities while still engaging Nrf2-related resilience pathways.

Metabolic disease and aging provide a different translational landscape. These conditions often feature low-grade chronic inflammation, impaired mitochondrial flexibility, and blunted adaptive stress responses, including attenuated Nrf2 signaling with age.^36^ Here, the goal may not be strong acute activation but long-term restoration of redox responsiveness. H_2_ has attracted attention because long-term intake appears feasible, while H_2_S donors may help preserve vascular and mitochondrial function if release kinetics are controlled.^29^,^37^ The Nrf2 axis is attractive in this context because it coordinates redox defense with intermediary metabolism rather than acting as a purely antioxidant endpoint.

Cardiovascular and endothelial disorders also illustrate the value of comparative thinking. Endothelial dysfunction reflects not only oxidant excess but also impaired NO bioavailability, inflammatory activation, and mitochondrial stress. H_2_S and NO interact directly in the vascular compartment, while H_2_ may preserve signaling competence by reducing oxidative consumption of NO-related intermediates under stress.^21^,^25^ If Nrf2 induction accompanies these effects, then vascular benefit may emerge from combined improvement in signaling tone, antioxidant capacity, and mitochondrial integrity rather than from any single gas action (**Table 2**).

**Table 2:** Translational opportunities and constraints for therapeutic gases in different diseases

Disease	Pathobiological problem most relevant to Nrf2	Therapeutic logic for gas selection	Potential role of combination strategy	Main translational limitation
Ischemia–reperfusion injury	Energetic collapse followed by reperfusion ROS/RNS burst and endothelial dysfunction	H_2_S for metabolic suppression/preconditioning; NO for perfusion and endothelial support; H_2_ for broad attenuation of oxidative propagation	Stage-specific use is plausible because ischemic and reperfusion phases are mechanistically distinct	Need precise timing and dose control to avoid hypotension, sulfide toxicity, or underdosing
Neurodegenerative disease	Chronic oxidative stress, mitochondrial dysfunction, neuroinflammation, blood–brain barrier access	H_2_ is attractive for systemic delivery and CNS penetration; targeted NO/H_2_S donor strategies may complement local redox signaling	Combination may be useful only with targeted delivery because the CNS is highly sensitive to off-target gas exposure	Systemic NO/H_2_S exposure may be limited by peripheral clearance and toxicity
Metabolic disease and aging	Blunted adaptive stress responses, impaired mitochondrial flexibility, chronic low-grade inflammation	Long-term low-risk H_2_ exposure and slow H_2_S release are conceptually aligned with restoration of Nrf2 responsiveness	Combination strategies may help if they restore vascular and mitochondrial adaptation without chronic overstimulation	Need sustained but sub-toxic dosing; chronic Nrf2 hyperactivation may not always be beneficial
Vascular dysfunction	Loss of NO bioavailability, endothelial oxidative stress, inflammation	NO/H_2_S crosstalk and H_2_-mediated buffering of severe oxidative conditions may preserve signaling competence	Cooperation is biologically plausible because the vascular compartment hosts direct NO-H_2_S interactions	Requires biomarkers that distinguish improved signaling from simple vasodilatory side effects

CNS: Central nervous system; H_2_: hydrogen; H_2_S: hydrogen sulfide; NO: nitric oxide; Nrf2: nuclear factor erythroid 2-related factor 2; RNS: reactive nitrogen species; ROS: reactive oxygen species.

## Clinical Translation of Nrf2-Centered Gas Framework

The main barrier to clinical translation is not conceptual scarcity but delivery control. Gases are small, diffusible, and often short-lived. That makes them attractive biologically but difficult pharmacologically. Inhalation can produce rapid systemic exposure but may be impractical or unsafe for some molecules and settings. Donor compounds can provide localized release but introduce by-products, release kinetics, and tissue specificity as additional variables.^30^,^37^,^38^

The dosing problem is particularly acute for NO and H_2_S. Both exhibit steep therapeutic windows. Too little exposure may fail to engage protective signaling; too much may worsen hypotension, mitochondrial inhibition, or nitrative injury.^8^,^18^ Gas therapy requires a concentration-time profile that is sufficient to trigger adaptive signaling but insufficient to collapse physiological gradients or generate toxic secondary chemistry. Nrf2 activation itself follows this logic: transient and appropriately scaled activation is often beneficial, whereas excessive or chronic activation can become maladaptive.^6^

H_2_ differs because its safety margin is broader, but this does not eliminate delivery questions. Concentration at target tissue, duration of dissolved H_2_, and route-specific kinetics still affect biological output. Hydrogen-rich water is convenient but variable in dose delivery; inhalation offers better control but different practical constraints; emerging carriers aim to prolong retention or localize release.^26^,^31^ Because H_2_ likely acts indirectly, timing relative to the evolving injury process may matter as much as absolute concentration.

Advanced delivery systems are therefore central to the future of the field. Nanocarriers, gas-releasing molecules, hybrid donor systems, and stimuli-responsive platforms are being developed to transform diffuse gas exposure into more programmable pharmacology.^38^,^39^ For NO and H_2_S, responsive systems may reduce burst toxicity while preserving signaling potency. For H_2_, carrier technologies may increase local residence time or allow combination with other therapeutics. In principle, these tools also enable sequence-specific multi-gas strategies, although that possibility remains underexplored (**Figure 5**).

**Figure 5:**
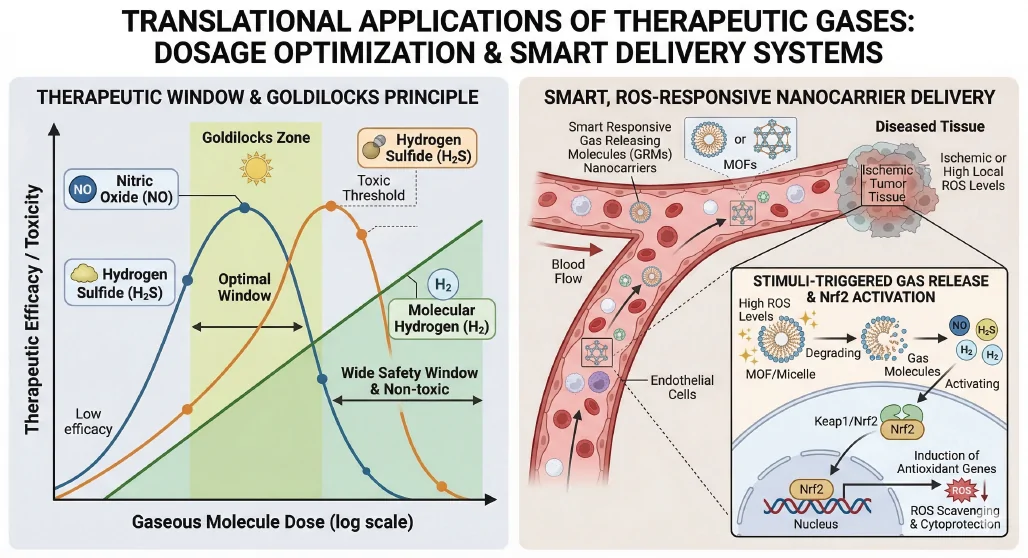
Translational framework of dose optimization, release control, and advanced delivery strategies for gas-based interventions. Created with BioRender.com. H_2_: Hydrogen; H_2_S: hydrogen sulfide; Keap1: Kelch-like Ech-associated protein 1; MOFs: metal-organic frameworks; NO: nitric oxide; Nrf2: nuclear factor erythroid 2-related factor 2; ROS: reactive oxygen species.

## Biomarkers, Mechanistic Bottlenecks, and Experimental Priorities

One reason mechanistic debates persist is that the field still lacks sufficiently precise *in vivo* readouts. Many studies infer gas action from downstream antioxidant enzyme expression, total glutathione, or bulk oxidative stress markers. These are useful but indirect. A stronger mechanistic program requires real-time or residue-resolved detection of S-nitrosylation, persulfidation, oxidized phospholipid mediators, and subcellular redox shifts in the same biological system.^39^,^40^

For Nrf2 specifically, researchers often stop at nuclear translocation or induction of HO-1, NQO1, or GCLC. These markers are important but not sufficient to distinguish canonical Keap1 cysteine sensing from noncanonical autophagy- or kinase-driven activation. Nor can they separate direct gas chemistry from secondary changes in mitochondrial function or inflammatory burden. Future work should therefore combine transcriptional readouts with residue-level Keap1 modification mapping, reporter systems for compartmental redox status, and experimental designs that manipulate upstream kinases or autophagy nodes in parallel.

Acute injury trials may prioritize organ-function recovery, inflammatory biomarkers, and mitochondrial injury markers. Chronic disease studies may need repeated measurements of metabolic flexibility, endothelial function, imaging, and transcriptomic adaptation. Because Nrf2 is a network regulator, success may be missed if evaluation focuses too narrowly on one antioxidant enzyme or one plasma oxidative marker. A biomarker strategy that reflects pathway breadth will better match the biology of gas-mediated adaptation. Comparative studies are harder to perform, but they are essential. Only by testing NO, H_2_S, and H_2_ side by side under matched injury models, matched delivery conditions, and matched pathway assays can researchers determine where convergence is real, where mechanisms diverge, and where combination logic is justified. A Nrf2-centered framework provides the structure for such comparisons, but careful experimentation must supply the evidence.

## Limitations

Most available evidence is preclinical, and substantial heterogeneity exists in experimental models, gas donors, delivery methods, doses, and outcome measures. Direct comparisons among NO, H_2_S, and H_2_ remain limited, while some proposed mechanisms, particularly those involving H_2_, are supported mainly by indirect evidence.

## Conclusion

NO, H_2_S, and H_2_ do not converge on the Nrf2/Keap1 axis by identical routes. NO and H_2_S are redox-active signaling molecules whose access to Keap1 is grounded in direct or near-direct cysteine chemistry, whereas H_2_ appears to influence the pathway mainly through indirect remodeling of oxidative propagation, mitochondrial function, and stress-responsive signaling networks.^11^,^19^,^22^ Recognizing this asymmetry is essential for accurate mechanistic interpretation.

At the same time, these differences do not negate convergence. The three gases repeatedly stabilize overlapping adaptive states characterized by improved antioxidant defense, preserved mitochondrial quality, reduced inflammatory amplification, and enhanced stress tolerance. Nrf2 is a central translator of these states because it connects thiol sensing to transcriptional programs governing detoxification, metabolism, proteostasis, and organelle homeostasis.^3^,^32^

The most useful conceptual advance is therefore to view therapeutic gases not as interchangeable antioxidants, but as members of an interacting gas signaling network with distinct dominant chemistries and partially shared downstream architecture. It also emphasizes that future progress depends less on rhetorical claims of synergy and more on disciplined work defining timing, dose windows, delivery systems, and mechanistic biomarkers.

**Open Questions**

1. Which Keap1 cysteines are functionally modified by NO and H_2_S *in vivo*, and how context dependent are these modifications?
2. Does molecular hydrogen have a defined primary molecular target, or does it act mainly through distributed redox and metabolic modulation?
3. Under what conditions does crosstalk among NO, H_2_S, and H_2_ produce genuine therapeutic synergy?

## Data Availability

*Not available.*
